# Endopeptidase Regulation as a Novel Function of the Zur-Dependent Zinc Starvation Response

**DOI:** 10.1128/mBio.02620-18

**Published:** 2019-02-19

**Authors:** Shannon G. Murphy, Laura Alvarez, Myfanwy C. Adams, Shuning Liu, Joshua S. Chappie, Felipe Cava, Tobias Dörr

**Affiliations:** a Department of Microbiology, Cornell University, Ithaca, New York, USA; b Weill Institute for Cell and Molecular Biology, Cornell University, Ithaca, New York, USA; c Laboratory for Molecular Infection Medicine, Department of Molecular Biology, Umeå University, Umeå, Sweden; d Department of Molecular Medicine, College of Veterinary Medicine, Cornell University, Ithaca, New York, USA; Fred Hutchinson Cancer Research Center

**Keywords:** Gram-negative, *Vibrio cholerae*, cell wall, hydrolase, metalloproteins, peptidoglycan, zinc starvation

## Abstract

The cell wall is a strong, yet flexible, meshwork of peptidoglycan (PG) that gives a bacterium structural integrity. To accommodate a growing cell, the wall is remodeled by both PG synthesis and degradation. Vibrio cholerae encodes a group of three nearly identical zinc-dependent endopeptidases (EPs) that are predicted to hydrolyze PG to facilitate cell growth. Two of these (ShyA and ShyC) are conditionally essential housekeeping EPs, while the third (ShyB) is not expressed under standard laboratory conditions. To investigate the role of ShyB, we conducted a transposon screen to identify mutations that activate *shyB* transcription. We found that *shyB* is induced as part of the Zur-mediated zinc starvation response, a mode of regulation not previously reported for cell wall lytic enzymes. *In vivo*, ShyB alone was sufficient to sustain cell growth in low-zinc environments. *In vitro*, ShyB retained its d,d-endopeptidase activity against purified sacculi in the presence of the metal chelator EDTA at concentrations that inhibit ShyA and ShyC. This insensitivity to metal chelation is likely what enables ShyB to substitute for other EPs during zinc starvation. Our survey of transcriptomic data from diverse bacteria identified other candidate Zur-regulated EPs, suggesting that this adaptation to zinc starvation is employed by other Gram-negative bacteria.

## Abstract (precis)

Bacteria encode a variety of adaptations that enable them to survive during zinc starvation, a condition which is encountered both in natural environments and inside the human host. In Vibrio cholerae, the causative agent of the diarrheal disease cholera, we have identified a novel member of this zinc starvation response, a cell wall hydrolase that retains function and is conditionally essential for cell growth in low-zinc environments. Other Gram-negative bacteria contain homologs that appear to be under similar regulatory control. These findings are significant because they represent, to our knowledge, the first evidence that zinc homeostasis influences cell wall turnover. Anti-infective therapies commonly target the bacterial cell wall; therefore, an improved understanding of how the cell wall adapts to host-induced zinc starvation could lead to new antibiotic development. Such therapeutic interventions are required to combat the rising threat of drug-resistant infections.

## INTRODUCTION

**FIG 1:**
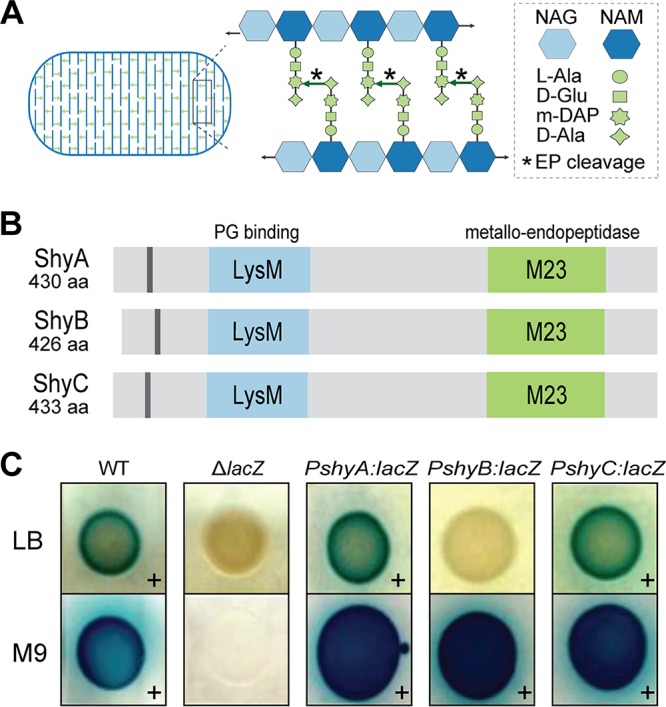
*shyB* encodes a LysM/M23 endopeptidase and is transcribed in minimal medium. (A) A model of the peptidoglycan sacculus, a polymer of β-(1,4)-linked *N*-acetylglucosamine (NAG) and *N*-acetylmuramic acid (NAM) glycan strands (blue). Cross-linked peptides (green arrows) and endopeptidase cleavage sites (*) are shown. m-DAP, *meso*-diaminopimelic acid. (B) The *V. cholerae* genome encodes three endopeptidases (ShyA, ShyB, and ShyC) possessing a hydrophobic region (gray), a PG binding domain (LysM, blue), and metallo-endopeptidase domain (M23, green). Protein domains were annotated using UniProt (64). aa, amino acids. (C) Promoter::*lacZ* transcriptional reporters for each endopeptidase were spotted onto LB (top row) and M9 (bottom row) agar containing X-Gal. A blue colony (+) indicates that the promoter is actively transcribed. Wild-type (WT) and *ΔlacZ* mutant strains are included as positive and negative controls, respectively.

The cell wall provides a bacterium with structural integrity and serves as a protective layer guarding against a wide range of environmental insults. Due to its importance for bacterial survival, the cell wall is a powerful and long-standing target for antibiotics (1). The wall is composed primarily of peptidoglycan (PG), a polymer of β-(1,4)-linked *N*-acetylglucosamine (NAG) and *N*-acetylmuramic acid (NAM) sugar strands (2) (Fig. 1A). Adjacent PG strands are linked to each other via peptide side chains, enabling the PG to assemble into a mesh-like structure called the sacculus (3). In Gram-negative bacteria, the sacculus is a single PG layer that is sandwiched between an inner and an outer membrane (4). This thin wall must be rigid enough to maintain cell shape and to contain high intracellular pressure (3, 5); however, the wall must also be flexible enough to accommodate cell elongation, cell division, and the insertion of transenvelope protein complexes (6). This requirement for both rigidity and flexibility necessitates continuous remodeling of the cell wall, which is accomplished by a delicate interplay between PG synthesis and degradation. Inhibition or dysregulation of either process can cause growth cessation or cell lysis, rendering the mechanisms of cell wall turnover an attractive target for new antibiotic development (7, 8).

PG synthesis is mediated by penicillin binding proteins (PBPs; the targets for beta-lactam antibiotics) and shape, elongation, division, and sporulation (SEDS) proteins (9). These proteins collectively catalyze cell wall synthesis through two main reactions, namely glycosyltransfer, to add new PG monomers to the glycan strand, and transpeptidation (TP) to crosslink the peptides of adjacent strands (2). Cell wall turnover is mediated by “autolysins,” a collective term for diverse and often redundant enzymes (amidases, lytic transglycosylases, and endopeptidases) that are able to cleave PG at almost any chemical bond (6). Endopeptidases (EPs), for example, hydrolyze the peptide crosslinks that covalently connect adjacent PG strands. EPs are crucial for cell elongation in several well-studied Gram-positive and Gram-negative rod-shaped bacteria (10–12), presumably because EPs create gaps in the PG meshwork to allow for the insertion of new cell wall material. Consistent with this proposed role, EP overexpression promotes PBP activity in Escherichia coli, likely through the generation of initiation sites for PG synthesis (13).

While EPs are essential for growth, they are also main drivers of PG degradation after inhibition of PBPs (14, 15). Thus, EP activity must be tightly controlled under normal growth conditions. EPs in two divergent bacterial species (E. coli and Pseudomonas aeruginosa) are proteolytically degraded to adapt to conditions that require changes in PG cleavage activity (16, 17), such as the transition into stationary phase. In Bacillus subtilis, EP expression is regulated by growth-phase dependent sigma factors (18–21). However, it is not known how EP expression is modified in response to specific environmental stresses.

In this study, we investigate the genetic regulation of specialized EPs in Vibrio cholerae, the causative agent of the diarrheal disease cholera. V. cholerae encodes three nearly identical EPs that are homologous to the well-characterized d,d-endopeptidase MepM in E. coli (10). Each EP contains a LysM domain that likely binds PG (22) and a putatively Zn^2+^-dependent M23 catalytic domain that hydrolyzes peptide cross-links (23) (Fig. 1B). We previously showed that two of these homologs (ShyA and ShyC) are housekeeping EPs that are collectively essential for V. cholerae growth (12). The gene encoding the third EP, *shyB*, is not transcribed under standard laboratory conditions (LB medium), and thus little is known about its biological function. To elucidate the role of ShyB, we conducted a transposon screen to identify mutations that promote *shyB* expression in LB. We found that *shyB* is induced by zinc starvation and, unlike the other two M23 EPs, ShyB enzymatic activity is resistant to treatment with the metal chelator EDTA. These data suggest that ShyB acts as an alternative EP to ensure proper PG maintenance during zinc starvation. Importantly, this represents the first characterization of an autolysin that is controlled by Zur-mediated zinc homeostasis and provides insight into how other Gram-negative bacteria might alter EP activity in zinc-limited environments.

## RESULTS

### *shyB* is repressed in LB but transcribed in minimal medium.

The hydrolytic activity of EPs needs to be carefully controlled to maintain cell wall integrity. We therefore considered it likely that specialized EPs are transcriptionally regulated and only induced when required. To test this hypothesis, we examined the expression patterns of *V. cholerae*’s LysM/M23 endopeptidases using *lacZ* transcriptional fusions. We first compared promoter activity on LB and M9 agar, as our previous work had shown that a Δ*shyB* mutation exacerbates a Δ*shyA* growth defect in M9 minimal medium (12). The *P_shyA_*::*lacZ* and *P_shyC_*::*lacZ* reporters generated a blue colony color on both LB and M9 agar (Fig. 1C); thus, these promoters are actively transcribed in either medium. Quantification of β-galactosidase (LacZ) activity in liquid culture showed that *P_shyA_* transcription does not vary between LB and M9 media, whereas *P_shyC_* promoter activity was slightly lower, yet still robust, in M9 minimal medium (Fig. S1). These data are consistent with ShyA and ShyC’s role as the predominant growth-promoting EPs (12). In contrast, *P_shyB_*::*lacZ* yielded blue colonies and produced detectable levels of β-galactosidase activity in M9 minimal medium only, indicating that the *shyB* promoter is induced in M9 medium but tightly repressed in LB (Fig. 1C and S1).

10.1128/mBio.02620-18.1 FIG S1 *shyA* and *shyC* transcriptional reporters are active in both LB and M9 minimal medium. *V. cholerae* C6706 carrying *P_shyA_*, *P_shyB_,* or *P_shyC_*::*lacZ* transcriptional fusions were grown from a single colony in 5 ml of LB, M9 minimal medium, or M9 medium plus ZnSO_4_ (500 nM) in a 30°C shaker. β-Galactosidase assays were performed (see details in Materials and Methods) with 0.5 ml of exponential-phase culture. Error bars represent the standard error of the mean (SEM) of three biological replicates. Statistical significance was measured using a one-way analysis of variance (ANOVA) followed by Dunnett’s multiple-comparison test (**, *P* < 0.01; n.s., *P* > 0.05). Download FIG S1, PDF file, 0.1 MB. Copyright © 2019 Murphy et al. 2019 Murphy et al. This content is distributed under the terms of the Creative Commons Attribution 4.0 International license.

### *shyB* is induced by zinc starvation.

**FIG 2:**
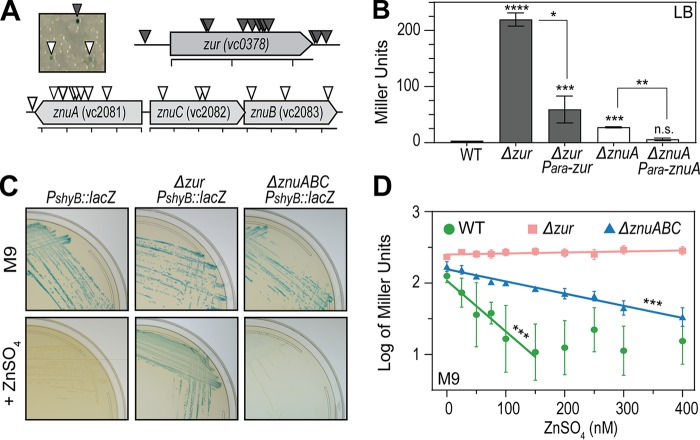
*shyB* transcription is regulated by zinc availability. (A) The *shyB* transcriptional reporter (*lacZ*::*P_shyB_*::*lacZ*) was mutagenized with a Himar1 mariner transposon and screened for *P_shyB_* induction (blue colonies) on LB agar containing X-Gal and selective antibiotics (see Materials and Methods). Representative transposon (Tn) mutants are shown. Insertion sites producing dark-blue colonies (gray triangles) and light-blue colonies (white triangles) were mapped using arbitrary PCR (scale bar = 200-bp increments). (B) *shyB* promoter activity was quantified using β-galactosidase assays (see Materials and Methods) in WT, Δ*zur* mutant, and Δ*znuA* mutant backgrounds. Each strain carries an arabinose-inducible plasmid (pBAD) that is either empty or complements the deleted gene in *trans*. Assays were conducted in LB containing chloramphenicol and arabinose (0.2%). Error bars represent the standard error of the mean (SEM) of three biological replicates. Statistical significance was measured using one-way analysis of variance (ANOVA) on natural log-transformed data, followed by Dunnett’s multiple-comparison test (****, *P* < 0.0001; ***, *P* < 0.001; **, *P* < 0.01; *, *P* < 0.05; nonsignificant [n.s.], *P* > 0.05). (C). The *shyB* transcriptional reporter in a WT, *Δzur* mutant, or *ΔznuABC* mutant background was grown on M9 X-Gal agar without (top row) or with (bottom row) 10 μM ZnSO_4_. (D) β-Galactosidase activity of the P*_shyB_*::*lacZ* reporter in a WT, *Δzur* mutant, or *ΔznuABC* mutant background was measured in M9 minimal medium supplemented with increasing concentrations of ZnSO_4_ (0 to 400 nM). Miller units were log-transformed and plotted against exogenous zinc concentration (nanomolar). The linear portions of the graph were fit with a regression line (WT, *R*^2^ = 0.95; *Δzur* mutant, *R*^2^ = 0.35; *ΔznuABC* mutant, *R*^2^ = 0.98), and asterisks indicate that slope of the regression is significantly nonzero. Error bars represent the SEM of three biological replicates.

To elucidate the specific growth conditions that favor *shyB* expression, we sought to identify the genetic factors regulating *shyB* transcription. To this end, we subjected the *shyB* transcriptional reporter strain to Himar1 mariner transposon mutagenesis and screened for *lacZ* induction (blue colonies) on LB agar. After two independent rounds of mutagenesis (50,000 total colonies), the screen yielded 26 blue insertion mutants. These were divided into two distinct classes according to colony color, with 12 dark-blue and 14 light-blue colonies. Strikingly, arbitrary PCR (24) mapped all 26 transposon insertions to only two chromosomal loci; both contained genes whose products play roles in zinc homeostasis, *vc2081-2083*–*znuABC* (light-blue colonies) and *vc0378*–*zur* (dark-blue colonies) (Fig. 2A). *znuABC* encodes V. cholerae*’*s high-affinity zinc uptake system (25), while Zur is a Fur family transcriptional regulator and the central repressor in the zinc starvation response (26). Under zinc-rich conditions, Zur and its Zn^2+^ corepressor bind to promoters containing a “Zur box” and block transcription (27). Under low-zinc conditions, Zur dissociates from promoters to induce the zinc starvation response (28). This response includes genes encoding zinc uptake systems (i.e., *znuABC* and *zrgABCDE*) (25) and zinc-independent paralogs that replace proteins that ordinarily require zinc for function (i.e., ribosomal proteins) (29).

To validate these transposon hits, we constructed clean deletions of *zur* and *znuA* in the P*_shyB_*::*lacZ* reporter strain. Deletion of either gene resulted in activation of the *shyB* promoter, as indicated by blue colony color on LB agar (Fig. S2A) or β-galactosidase activity measured in LB broth (Fig. 2B). P*_shyB_* repression was restored by expressing the deleted genes in *trans* (Fig. 2B and S2A). Thus, *shyB* is induced under conditions that are expected to either mimic (*zur* inactivation) or impose [*znuA*(*BC*) inactivation] zinc starvation.

10.1128/mBio.02620-18.2 FIG S2 The *shyB* promoter is induced by *Δzur* and *ΔznuABC* mutations on LB agar and repressed by exogenous zinc addition on M9 agar. (A) Clean deletions of *Δzur* and *ΔznuA* in *V. cholerae* N16961 carrying the *ΔlacZ*::*P_shyB_*::*lacZ* transcriptional reporter were complemented with an arabinose-inducible (pBAD) plasmid that is either empty or carries the respective gene in *trans*. Strains were plated onto LB agar containing X-Gal (40 μg ml^−1^), chloramphenicol (10 μg ml^−1^), and arabinose (0.2%). (B) *V. cholerae* N16961 carrying the *ΔlacZ*::*P_shyB_*::*lacZ* transcriptional reporter was plated on M9 X-Gal (40 μg ml^−1^) agar containing 10 μM of ZnSO_4_, FeSO_4_, or MnCl_2_. (A and B) All plates were incubated overnight at 30°C and then at room temperature for 2 days. Download FIG S2, JPG file, 0.2 MB. Copyright © 2019 Murphy et al. 2019 Murphy et al. This content is distributed under the terms of the Creative Commons Attribution 4.0 International license.

If zinc starvation is the factor inducing *shyB* expression in M9 medium, we would expect the P*_shyB_*::*lacZ* reporter to be repressed by external zinc addition. Indeed, supplementing M9 agar plates with 10 µM ZnSO_4_ was sufficient to turn off the *shyB* promoter in a wild-type (WT) background (Fig. 2C), whereas repression could not be achieved by the addition of other transition metals (iron and manganese) (Fig. S2B). Zinc supplementation repressed the reporter in a Δ*znuABC* mutant but not in a Δ*zur* mutant (Fig. 2C). This suggests that the P*_shyB_* activation in the Δ*znuABC* mutant is caused by zinc deficiency, while activation in the Δ*zur* mutant is due to the loss of zinc-sensing repression mechanism. To quantify the effect of zinc on *shyB* promoter activity, we grew the WT, Δ*zur* mutant, and Δ*znuABC* mutant reporter strains in M9 medium supplemented with increasing concentrations of zinc and measured β-galactosidase activity. As expected, P*_shyB_*::*lacZ* expression in WT and in Δ*znuABC* mutant tapered off at higher zinc concentrations, whereas expression in the Δ*zur* mutant was zinc independent (Fig. 2D). These data demonstrate that *shyB* transcription is repressed by high zinc availability and that the repression mechanism requires Zur.

### Zur directly binds the *shyB* promoter.

**FIG 3:**
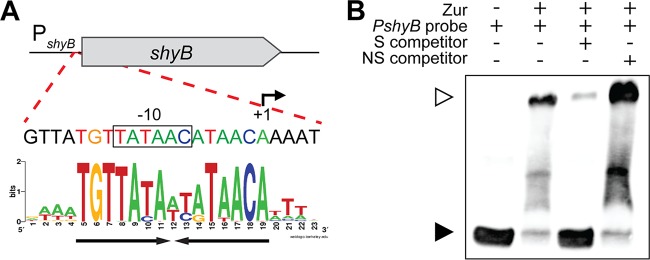
Zur directly binds the *shyB* promoter. (A) The *shyB* promoter, annotated with a 5′-RACE transcription start site (+1) and putative −10 region (box), was aligned with a *Vibrio* Zur sequence logo (30, 31). The inverted AT-rich repeat in the putative Zur box is underlined with black arrows. (B) A chemiluminescent probe containing the putative *shyB* Zur box was incubated with purified Zur in the presence of ZnCl_2_ (5 µM). Zur binding specificity was tested by adding 100-fold molar excess of unlabeled specific (S, lane 3) or nonspecific (NS, lane 4) competitor DNA. Samples were electrophoresed on a 6% DNA retardation gel to separate unbound (black arrow) and bound probe (white arrow).

Given Zur’s well-defined role as a zinc-sensing transcriptional regulator (27) and its requirement for *P_shyB_* repression in zinc-rich media, we hypothesized that Zur directly binds the *shyB* promoter. To test this, we retrieved a Zur box sequence logo built from 62 known regulatory targets in the *Vibrionaceae* family (30, 31) and aligned it with the *shyB* promoter region. This alignment identified a highly conserved Zur box, which is characterized by an inverted AT-rich repeat (Fig. 3A). We used 5′-rapid amplification of cDNA ends (RACE) to locate the *shyB* transcriptional start site (tss) (+1) and found that the putative Zur box overlaps with both the −10 region and the tss. A bound Zur-Zn^2+^ complex at this position likely prevents RNA polymerase binding and thereby prevents transcription (32).

To determine if Zur binds the *shyB* promoter *in vitro*, we then incubated purified Zur with a labeled DNA probe encoding the *P_shyB_* Zur box. Binding was assessed in the presence of ZnCl_2_ using an electrophoretic mobility shift assay (EMSA). As evident by a band shift, Zur forms a complex with the P*_shyB_* DNA *in vitro* (Fig. 3B, lanes 1 and 2). To examine DNA binding specificity, a 100-fold molar excess of unlabeled specific (S) or nonspecific (NS) competitor DNA was included in the binding reaction. The S competitor, which carries a sequence identical to that of the labeled probe, effectively sequestered Zur and increased the amount of unbound labeled probe (Fig. 3B, lane 3). Meanwhile, the NS competitor was ineffective at binding Zur (Fig. 3B, lane 4). These data indicate that the *shyB* promoter contains an authentic Zur box, and we conclude that *shyB* is a novel member of the Zur regulon.

### ShyB supports growth in chelator-treated medium.

As *shyB* is part of the Zur-mediated zinc starvation response, we hypothesized that V. cholerae relies on ShyB endopeptidase activity when zinc availability is low. To induce zinc starvation and robustly derepress the Zur regulon, V. cholerae strains were grown in M9 minimal medium supplemented with TPEN [*N,N,N′,N′*-tetrakis(2-pyridylmethyl)ethylenediamine], a cell-permeable metal chelator with high affinity for zinc (33). As expected from our genetic analysis, TPEN addition resulted in the production of ShyB protein (as measured by Western blotting), which could be reversed by adding zinc (Fig. S3A).

10.1128/mBio.02620-18.3 FIG S3 Western blots comparing ShyB and ShyC protein levels in M9 minimal or LB medium. (A) N16961 strains encoding tagged chromosomal versions of ShyB ΔLysM::His_6_-FLAG (sandwich fusion) or ShyC-His_6_-FLAG (C-terminal fusion) were grown in M9-glucose (0.4%) with added TPEN (250 nM) or TPEN plus ZnSO_4_ (1 µM). Cells were harvested at mid-log phase (OD_600_, 0.4) and lysed via SDS boiling and sonication. Western blotting was performed using standard techniques. Blots were developed using a mouse anti-FLAG F1804 primary antibody (Sigma-Aldrich) and goat anti-Mouse IR CW800 secondary antibody (LI-COR Biosciences). Blots were imaged using a Lycor Odyssey CLx imager. M, standard protein marker. (B) N16961 strains with tagged chromosomal versions of ShyA ΔLysM::His-FLAG (lane 1), ShyB ΔLysM::His-FLAG (lane 2), and ShyC-His-FLAG (lane 3) were grown in LB and harvested at mid-log phase (OD_600_, 0.5). Lane 4 shows ShyB ΔLysM::His-FLAG in a Δ*zur* background. Western blotting was performed as described above. Download FIG S3, PDF file, 0.7 MB. Copyright © 2019 Murphy et al. 2019 Murphy et al. This content is distributed under the terms of the Creative Commons Attribution 4.0 International license.

**FIG 4:**
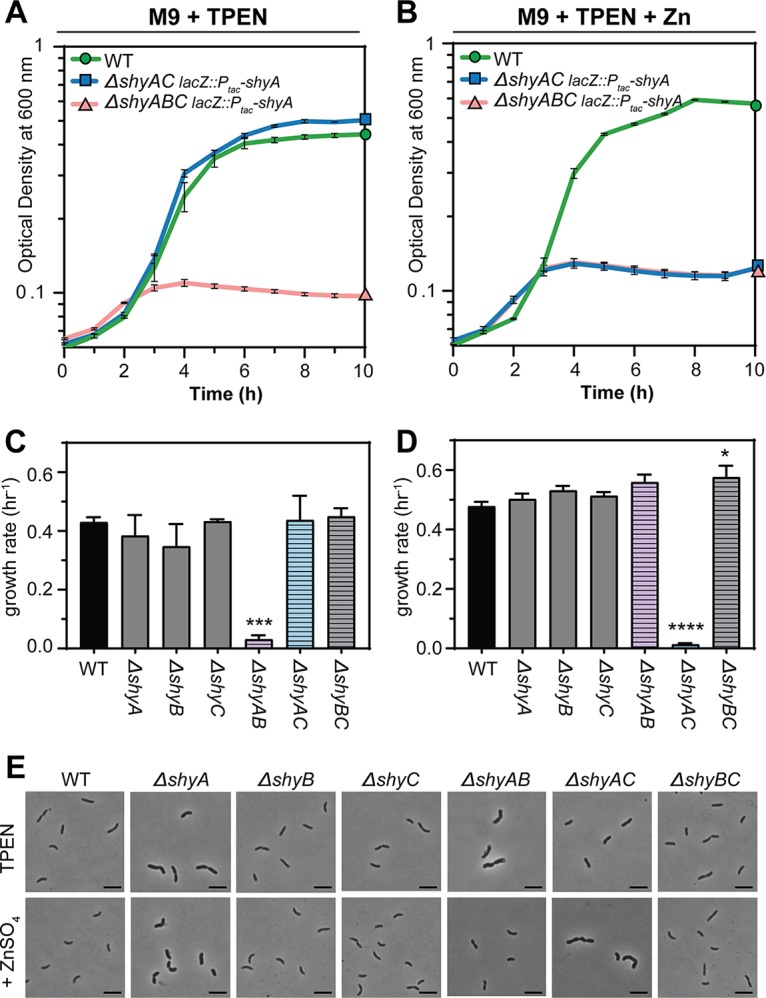
ShyB supports cell growth and is conditionally essential in a *ΔshyA* mutant in TPEN-treated medium. Mid-exponential cultures of the indicated *V. cholerae* mutants were washed to remove IPTG before being diluted 1:100 into M9-glucose-streptomycin plus TPEN (250 nM) in the absence (A and C) or presence (B and D) of ZnSO_4_ (1 μM). Growth of each strain was monitored by the optical density at 600 nm in a Bioscreen C 100-well plate. Error bars report standard error of the mean (SEM) for three independent biological replicates. (A and B) Growth curves on a log scale are shown for WT (green circle), *ΔshyAC lacZ*::*P_tac_*-*shyA* mutant (blue square), and *ΔshyABC lacZ*::*P_tac_*-*shyA* mutant (red triangle). (C and D) Growth rates (per hour) of WT (solid black), single EP mutants (solid gray), and double EP mutants (striped) were calculated from changes in optical density during exponential phase. Error bars report the SEM for three independent biological replicates. Statistical difference relative to the WT was assessed using a one-way ANOVA followed by Dunnett’s multiple-comparison test (****, *P* < 0.0001; ***, *P* < 0.001; *, *P* < 0.05). (E) Phase-contrast images of *V. cholerae* (C and D) sampled from mid-log phase (scale bar = 5 μM).

We first tested whether native *shyB* could restore growth to the Δ*shyAC* mutant under zinc starvation conditions. *shyA* and *shyC* deletions were generated in a parent strain expressing an isopropyl thio-β-d-galactopyranoside (IPTG)-inducible copy of *shyA* (*lacZ*::*P_tac_*-*shyA),* as these genes are conditionally essential in rich medium (12). In the absence of IPTG, we found that chelation with either TPEN or EDTA (a more general divalent metal ion chelator) induced growth of the Δ*shyAC* mutant but not in the mutant that additionally lacked *shyB* (Fig. 4A and S4A). As expected, chelation-dependent growth of the Δ*shyAC* mutant could be suppressed by adding zinc (Fig. 4B and S4A). These data suggest that induction of *shyB* alone is sufficient to sustain V. cholerae growth, and synthetic lethality of *shyA* and *shyC* is due to the lack of *shyB* expression under laboratory growth conditions. Consistent with this interpretation, exogenous *shyB* expression restored growth to the Δ*shyABC P_tac_-shyA* mutant (Fig. S4B), and we were able to generate a Δ*shyAC* knockout in a Δ*zur* background that grows robustly in LB medium (Fig. S4C).

10.1128/mBio.02620-18.4 FIG S4 *shyB* expression driven by chelation, induction, or zur deletion restores growth to the *ΔshyAC* mutant. (A and B) Strains were grown overnight in LB-streptomycin plus IPTG (200 μM) at 37°C. Cells were washed, subcultured 1:10 into M9-glucose (0.4%), and grown at 37°C for 2 h. (A) EDTA-chelation restores growth to a *ΔshyAC* mutant. WT (green), *ΔshyAC lacZ*::*P_tac_-shyA* mutant (blue), and *ΔshyABC lacZ*::*P_tac_-shyA* mutant (red) strains were diluted 1:100 into M9-glucose (0.4%) containing EDTA (30 µM) (solid lines) or EDTA plus ZnSO_4_ (60 μM) (dashed lines). (B) Exogenous *shyB* expression supports growth in a *ΔshyABC lacZ*::*P_tac_-shyA* mutant. WT (dotted lines) and *ΔshyABC lacZ*::*P_tac_-shyA* vc1807::*P_ara_-shyB* mutant (solid lines) strains were diluted 1:100 in M9-glucose (0.4%) (orange), with 200 µM IPTG (green) or with 0.2% arabinose (black). (C) *zur* deletion restores growth to a *ΔshyAC* mutant in LB medium. Overnight cultures (grown in LB-streptomycin at 37°C) were subcultured 1:100 into fresh medium and grown at 37°C for 2 h. *Δzur lacZ*::*P_tac_-zur* (blue) and *Δzur ΔshyAC lacZ*::*P_tac_-zur* (red) mutants were diluted 1:100 into LB (solid lines) or in LB plus IPTG (200 µM) (dashed lines). (A to C) Growth of each 200-µl culture was measured by optical density (at 600 nm) in a Bioscreen C 100-well plate. Error bars report standard error of the mean (SEM) for three biologically independent replicates. Download FIG S4, PDF file, 0.6 MB. Copyright © 2019 Murphy et al. 2019 Murphy et al. This content is distributed under the terms of the Creative Commons Attribution 4.0 International license.

A *shyB* deletion alone did not result in a significant defect in growth rate or morphology in TPEN-treated M9 medium (Fig. 4C and E); however, autolysins often need to be deleted in combination to result in a substantial phenotype (11). We therefore generated all possible combinations of LysM/M23 endopeptidase deletions to broadly dissect the relevance of zinc concentrations for EP activity. *ΔshyA* mutant cells were somewhat enlarged under either growth condition but did not exhibit a strong growth rate defect (Fig. 4C to E). The Δ*shyAB* double mutant failed to grow in TPEN-treated M9 medium (Fig. 4C), and cells exposed to this condition were aberrantly thick and long (Fig. 4E). This indicates that ShyC, the only essential LysM/M23 EP in the Δ*shyAB* mutant, cannot support growth in zinc-starved medium. In zinc-replete medium, the Δ*shyAC lacZ*::*P_tac_*-*shyA* mutant failed to grow in the absence of IPTG and displayed a similar aberrant cell morphology (Fig. 4D and E), consistent with a lack of *shyB* expression under this condition. This trade-off in synthetic lethality partners in low-zinc (Δ*shyAB* mutant) and high-zinc (Δ*shyAC*) growth media tentatively suggests that ShyB may function as a replacement for ShyC during zinc starvation. ShyC protein levels, as measured by Western blotting, were not reduced in the presence of TPEN (Fig. S3A), ruling out the possibility that Δ*shyAB* mutant lethality reflects downregulation or degradation of ShyC. Rather, these observations suggest that ShyC activity is more sensitive to zinc chelation than the other EPs. Alternatively, TPEN might induce changes in PG architecture that make it resistant to cleavage by ShyC.

### ShyB is an EDTA-resistant d,d-endopeptidase *in vitro*.

**FIG 5:**
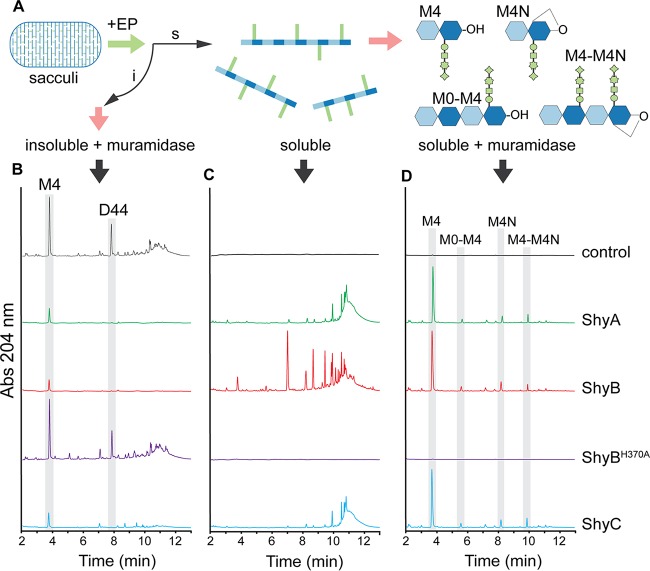
ShyB is a d,d-endopeptidase *in vitro*. (A) *V. cholerae* sacculi were digested with 10 µg of endopeptidase (ShyA, ShyB, ShyB^H370A^, and ShyC) or a no-enzyme control for 16 h at 37°C. The sample was separated into insoluble (i) and soluble (s) fractions, and each component was further digested with muramidase (pink arrows). The digestion products were separated by ultrahigh-performance liquid chromatography (UPLC) and quantified by absorbance (Abs) at 204 nm. The chromatograms show the insoluble fraction digested with muramidase (B), the soluble fraction (C), and the soluble fraction digested with muramidase (D). Highlighted peaks indicate D44 (disaccharide tetratetrapeptide), M4 (monomer disaccharide tetrapeptide), M4N (anhydrous monomer disaccharide tetrapeptide) muropeptides, and oligomonomeric chains.

ShyB is predicted to be a zinc-dependent d,d-endopeptidase, but biochemical evidence is lacking. Thus, we measured the *in vitro* hydrolytic activity of each EP against V. cholerae sacculi. Recombinant Shy proteins were purified without the N-terminal signal sequence (ShyA_Δ1–35_, ShyB_Δ1–34_, and ShyC_Δ1–33_) (Fig. S5); as a negative control, we purified ShyB_Δ1–34_ with a mutation (H370A) in the active site that is expected to abolish activity. Each EP was incubated with purified V. cholerae sacculi; the soluble PG fragments released by digestion, as well as the remaining insoluble pellet, were treated with muramidase to process long glycan strands into their subunits (Fig. 5A). The resulting muropeptides were then separated using ultrahigh-performance liquid chromatography (UPLC) and quantified by spectrophotometry (see Materials and Methods for details).

10.1128/mBio.02620-18.5 FIG S5 SDS-PAGE gel of purified Shy proteins. Purified recombinant proteins ShyA_Δ1–35_ (A), ShyB_Δ1–34_ (B), ShyC_Δ1–33_ (C), and ShyB^H370A^_Δ1–34_ (D) were run on a SDS-PAGE gel and stained with Coomassie blue. Each lane represents a greater fold dilution of the purified protein. M, standard protein marker. Download FIG S5, PDF file, 3.0 MB. Copyright © 2019 Murphy et al. 2019 Murphy et al. This content is distributed under the terms of the Creative Commons Attribution 4.0 International license.

The muramidase-digested insoluble fraction contains PG that was not released by EP digestion (Fig. 5B). The no-enzyme control showed a large peak corresponding to D44 dimers, indicating that these peptide crosslinks are abundant in the V. cholerae sacculus substrate (Fig. 5B). This D44 peak was noticeably absent from the ShyA-, ShyB-, and ShyC-digested sacculi. Instead, each of the Shy endopeptidases (but not the H370A mutant) hydrolyzed sacculi and generated a profile of soluble fragments (Fig. 5C). ShyA and ShyC produced similar muropeptide profiles, indicating similar hydrolytic activity *in vitro*, while the ShyB chromatogram revealed more peaks with shorter retention times (Fig. S6). Tandem mass spectrometry (MS/MS) analysis determined that these ShyB-generated peaks correspond to un-cross-linked oligo-NAG-NAM-tetrapeptide chains [M4]_2–4,_ as well as small amounts of M4, M4N, and M0–M4 monomers. Consistent with a differential cleavage activity, ShyB was able to further process PG predigested with ShyA or ShyC, while these EPs only slightly modified ShyB-digested PG (Fig. S7).

10.1128/mBio.02620-18.6 FIG S6 Identification of muropeptides released by ShyB digestion of whole *V. cholerae* sacculi. (A) Chromatogram showing the soluble products released by ShyB digestion (same as Fig. 5C). (B) Table of identified muropeptide peaks. Muropeptide identity was confirmed by MS/MS analysis, using a Xevo G2-DX Q-TOF system (Waters Corporation, USA). The difference in theoretical and observed monoisotropic masses (in grams per mole) was computed for each peak. Download FIG S6, PDF file, 0.2 MB. Copyright © 2019 Murphy et al. 2019 Murphy et al. This content is distributed under the terms of the Creative Commons Attribution 4.0 International license.

10.1128/mBio.02620-18.7 FIG S7 Sequential and time-dependent digestion of *V. cholerae* sacculi by Shy endopeptidases. Ten micrograms of purified ShyA (A), ShyB (B), and ShyC (C) was incubated with *V. cholerae* sacculi for 16 h at 37°C, followed by secondary digestion with a different endopeptidase. The soluble products released by digested sacculi were separated by UPLC and quantified by absorbance at 204 nm. In a similar experiment, analysis of the soluble muropeptides generated by EP activity was conducted at 1-h (D), 6-h (E), and 16-h (F) time points. Download FIG S7, PDF file, 0.3 MB. Copyright © 2019 Murphy et al. 2019 Murphy et al. This content is distributed under the terms of the Creative Commons Attribution 4.0 International license.

The soluble EP digestion products were further treated with muramidase (Fig. 5D). Each of the EP-treated samples contained a large peak near 4 min, corresponding to a M4 monomer, in addition to smaller amounts of M4N monomer and chains of monomers (M0–M4 and M4–M4N). These peaks were absent from the negative controls (no-enzyme and ShyB H370A). There was virtually no D44 peak detected in the Shy-treated samples, indicating that all three LysM/M23 EPs function as a d,d-endopeptidase *in vitro* (Fig. 5B). We speculate that the apparent unique activity of ShyB on whole sacculi may reflect its ability to process substrate with a more diverse set of conformations than ShyA and ShyC.

**FIG 6:**
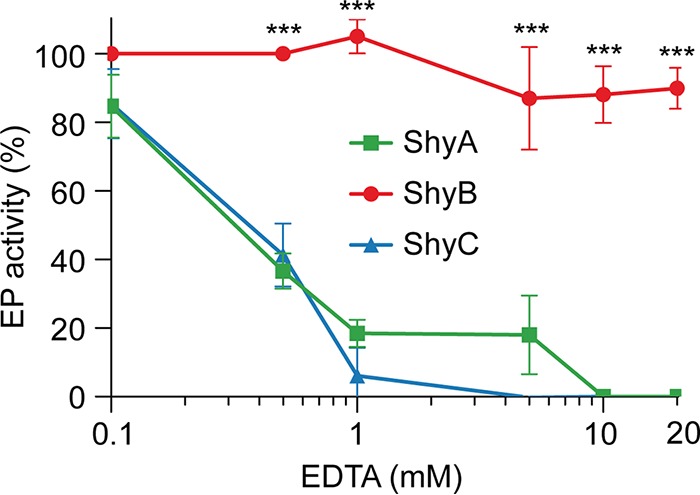
ShyB retains endopeptidase activity in the presence of EDTA *in vitro*. *V. cholerae* sacculi were digested with 10 µg of purified ShyA, ShyB, or ShyC for 16 h at 37°C in the absence or presence of EDTA, with concentrations ranging from 0.1 mM to 20 mM. The soluble products released by digested sacculi were separated by UPLC and quantified by absorbance at 204 nm. EP activity was measured by integrating the chromatogram profile of muramidase-digested insoluble fraction (as shown in Fig. 5B) and normalizing to the no-EDTA treatment (100% activity) and no-enzyme control (0% activity). Error bars represent the SEM of at least two biological replicates, and statistical significance was assessed using a two-way ANOVA followed by Dunnett’s multiple-comparison test (***, *P* < 0.0001).

M23 domains typically require a coordinated zinc ion to carry out PG hydrolysis (23). We have previously demonstrated that ShyA requires zinc for activity *in vitro* (12), and others have modeled zinc in the active site of a ShyB crystal structure (34). Based on its regulation by Zur, we hypothesized that ShyB evolved to function in zinc-limited environments. To test this, we repeated the *in vitro* PG hydrolysis assays under metal-limited conditions by using the divalent cation chelator EDTA. Strikingly, ShyB EP activity was largely unaffected by EDTA at the wide range of concentrations tested (0.1 mM to 20 mM) (Fig. 6 and Fig. S8). ShyA and ShyC had reduced activity in 0.5 mM EDTA and suffered a total loss of activity at higher concentrations, consistent with results previously obtained for ShyA (12). ShyC activity appeared to be more sensitive to EDTA than was ShyA (at 1 and 5 mM), but this difference was not statistically significant. These *in vitro* assays suggest that ShyB has a high affinity for, or can function without, divalent cations like zinc.

10.1128/mBio.02620-18.8 FIG S8 ShyB retains endopeptidase activity in the presence of EDTA *in vitro*. Ten micrograms of purified ShyA, ShyB, and ShyC was incubated with *V. cholerae* sacculi for 16 h at 37°C across a range of EDTA concentrations (0 mM to 20 mM). Muropeptides were separated by UPLC and quantified by absorbance at 204 nM (see Materials and Methods for details). The chromatograms for the soluble fraction (A to C) and the muramidase-digested insoluble pellet (D to F) for ShyA-treated (A and D), ShyB-treated (B and E), or ShyC-treated (C and F) sacculi are shown. Download FIG S8, PDF file, 2.0 MB. Copyright © 2019 Murphy et al. 2019 Murphy et al. This content is distributed under the terms of the Creative Commons Attribution 4.0 International license.

### Zur-regulated endopeptidases are widespread in divergent bacteria.

**FIG 7:**
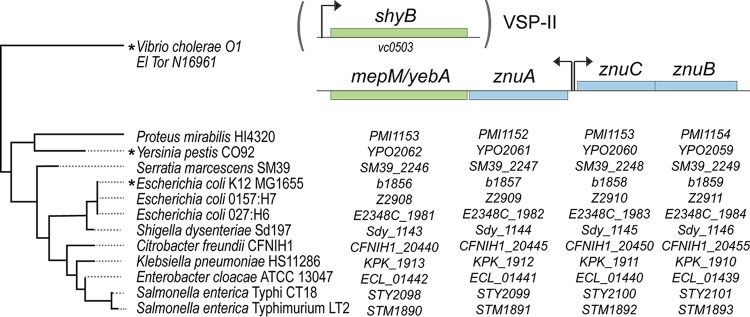
*shyB* exists as a single gene in *V. cholerae* N16961, while other pathogenic Gram-negative bacteria encode a ShyB homolog adjacent to the Zur-controlled *znu* operon. Gene neighborhood alignments, generated using the Prokaryotic Sequence homology Analysis Tool (PSAT) (65), show the arrangement of a LysM/M23 endopeptidase gene (*mepM/yebA*, green) and adjacent zinc importer genes (*znuABC*, blue) in 12 Gram-negative organisms. *shyB,* in *V. cholerae* O1 El Tor N16961, exists as a single gene on a *Vibrio* seventh pandemic island (VSP-II), separate from the *znuABC* locus. Arrows indicate the approximate location of the unidirectional or bidirectional promoter and site of Zur binding. Asterisks signify published data sets that support Zur- and/or zinc-dependent regulation of the endopeptidase. Evolutionary history of the species shown was inferred using alignments of 16S rRNA genes (66) and the neighbor-joining method for tree construction (67) in MEGA7 (68). Evolutionary distances were computed using the maximum composite likelihood method (69) and are in units of number of base substitutions per site.

Zur-regulated EPs appear to be widespread in the Vibrio genus. Using BLAST homology searches, we have identified isolates from 30 different non-cholerae Vibrio species that contain a ShyB homolog with a Zur box directly upstream of the open reading frame (Table S1) (35). To assess the significance of zinc homeostasis for EP regulation more broadly, we surveyed published microarray and RNA sequencing (RNA-seq) data sets from diverse bacteria for differential EP expression (36–45). Yersinia pestis CO92, the causative agent of plague, encodes a ShyB homolog (YebA, YPO2062) whose gene is significantly upregulated in a Δ*zur* mutant (36). The *yebA* gene does not contain its own Zur box but is positioned adjacent to *znuA* and may thus be cotranscribed as part of the same operon (Fig. 7). Similarly, *mepM* (b1856) is located adjacent to the *znu* locus in laboratory (K-12 MG1655) and pathogenic (enterohemorrhagic O157:H7 and enteropathogenic O127:H6) E. coli strains. Two microarray studies in E. coli, one of which was validated by quantitative PCR (qPCR), showed that *mepM* is transcriptionally upregulated in response to zinc starvation (44, 45). Notably, this *znu*-EP arrangement is conserved in many other Gram-negative pathogens, including Salmonella enterica (STM1890, STY2098), Enterobacter cloacae (ECL_01442), Klebsiella pneumoniae (KPK_1913), Shigella dysenteriae (Sdy_1143), Citrobacter freundii (CFNIH1_20440), Serratia marcescens (SM39_2246), and Proteus mirabilis (PMI1153) (Fig. 7). Collectively, these data suggest that zinc homeostasis and cell wall turnover may be linked in many Gram-negative bacteria.

10.1128/mBio.02620-18.9 TABLE S1 Summary of ShyB homologs that contain an upstream, canonical Zur box. Download Table S1, PDF file, 0.02 MB. Copyright © 2019 Murphy et al. 2019 Murphy et al. This content is distributed under the terms of the Creative Commons Attribution 4.0 International license.

## DISCUSSION

### Functionally redundant endopeptidases support cell growth.

The importance of EPs has been established in both Gram-negative and Gram-positive bacteria (10–12), supporting the long-standing hypothesis that autolysins create space in the PG meshwork for the insertion of new cell wall material (8). As with other autolysins, EPs are often functionally redundant under laboratory growth conditions but exhibit slight differences in cellular localization (12, 20, 46), substrate specificity (10, 47), and relative abundance during each growth phase (11, 46). Our previous work in V. cholerae identified three LysM/M23 zinc metallo-endopeptidases; two (ShyA and ShyC) are housekeeping enzymes that are conditionally essential for growth, while the role of the third (ShyB) has remained unknown (12). In this study, we define *shyB* as a new member of the Zur regulon and demonstrate that ShyB can replace the other EPs *in vivo* when derepressed by zinc limitation. This is a novel mechanism for regulating autolysins and establishes a link between two essential processes, cell wall turnover and metal ion homeostasis.

### Zinc availability affects the expression and activity of cell wall hydrolases.

We initially observed that the *shyB* promoter is active on M9 medium and repressed on LB agar. LB contains ∼12.2 μM zinc (48), while presumably the concentrations are much lower for the defined M9 medium (which does not contain any added zinc). Consistent with a role for the *zur* regulon in *shyB* regulation, we found that supplementing M9 medium with nanomolar concentrations of zinc was sufficient to repress the *shyB* promoter in liquid culture (Fig. 2D**)**. As a cautionary note, this suggests that V. cholerae is starved for zinc in M9 medium (and possibly other minimal media as well), a complication not usually considered when interpreting results obtained in this medium.

Based on its membership in the Zur regulon, it is likely that ShyB evolved to function in low-zinc environments. Indeed, ShyB endopeptidase activity *in vitro* is unaffected by EDTA [*K_d_*_(Zn2+)_ = 10^−16^ M] (*K_d_*, dissociation constant) (49), even at chelator concentrations (e.g., 20 mM) that induce levels of metal starvation that far exceed those encountered in nature (Fig. 6). We found that a mutation in a metal-coordinating residue (H370A) abolished ShyB activity (Fig. 5), but further biochemical characterization of the active site is required to explain how ShyB retains EP activity in low-zinc environments. It is possible that the ShyB active site (i) binds zinc with very high affinity, (ii) utilizes an alternative metal cofactor, or (iii) functions independently of a bound metal cofactor.

ShyA appears to have an intermediate ability to function in low-zinc environments. The ability to sustain growth in the presence of TPEN or EDTA indicates that ShyA function is less affected by metal starvation than ShyC; however, ShyA’s activity can still be inhibited by higher concentrations of EDTA *in vitro*. Collectively, our data suggest a model where ShyA is the predominant housekeeping endopeptidase. ShyC appears to partially overlap in function with ShyA when zinc availability is high. During zinc starvation, we speculate that *shyB* is derepressed specifically to compensate for a loss of ShyC activity.

ShyB can function as the sole EP under both high- and low-zinc conditions (Fig. 4A and S5), so why is it repressed under normal growth conditions rather than just replacing ShyA and ShyC altogether? One possible explanation is that ShyB activity might be more destructive than the other EPs, requiring more careful control. Indeed, our activity assays demonstrated that ShyB has a slightly altered activity profile, processing the sacculus into smaller fragments with shorter retention times (Fig. 5C) and doing so at a higher rate (Fig. S7D to F). Additionally, native ShyB protein levels (constitutively transcribed in a Δ*zur* mutant) are substantially lower than those of either ShyA or ShyC (Fig. S3B), suggesting that less enzyme is required to support growth.

### Zur-regulated endopeptidases are present in divergent Gram-negative bacteria.

Zur-regulated EPs are a novel adaptation to zinc limitation; however, ShyB activity is not essential to wild-type V. cholerae growth under the laboratory conditions tested. This might be due to functional overlap with the other Shy EPs, but it should also be noted that pandemic V. cholerae horizontally acquired *shyB* on a pathogenicity island (VSP-II) recently in its evolutionary history (in the 1960s) (50). It is thus possible that ShyB activity is of advantage under conditions that also positively selected for acquisition of VSP-II, such as pathogenesis.

Importantly, Zur-regulated EPs are not confined to *Vibrionaceae*, as divergent Gram-negative bacteria encode conserved ShyB/MepM/YebA homologs adjacent to the Zur-controlled *znu* operon (Fig. 7). Transcriptomic data from both Y. pestis and E. coli support the prediction that this EP is upregulated along with the zinc importer, though it is unclear whether these EPs can also be transcribed from a Zur-independent promoter internal to *znuA*. Parts of the zinc starvation response (e.g., zinc importers) have been shown to be required for host colonization by divergent human pathogens (51–54), since vertebrates and other hosts sequester metals as a form of nutritional immunity (55). It has also been shown that ShyB homolog YebA in Y. pestis is important for virulence in the plague pathogen (56). It is therefore tempting to speculate that the regulation of endopeptidase activity in low-zinc environments may play a general role in pathogenesis.

## MATERIALS AND METHODS

### Bacterial growth conditions.

Cells were grown by shaking (200 rpm) at 37°C in 5 ml of LB medium unless otherwise indicated. M9 minimal medium with glucose (0.4%) was prepared with ultrapure Milli-Q water to minimize zinc contamination. When appropriate, antibiotics were used at the following concentrations: streptomycin, 200 µg ml^−1^; ampicillin, 100 µg ml^−1^; kanamycin, 50 µg ml^−1^; and chloramphenicol, 5 µg ml^−1^. IPTG (200 µM) was added to all liquid and solid media if required to sustain V. cholerae growth. 5-Bromo-4-chloro-3-indolyl-β-d-galactopyranoside (X-Gal; 40 µg ml^−1^) was added to plates for blue-white screening.

### Plasmid and strain construction.

All genes were PCR amplified from V. cholerae El Tor N16961 genomic DNA. Plasmids were built using isothermal assembly (57) with the oligonucleotides summarized in Table S2. The suicide vector pCVD442 was used to make gene deletions via homologous recombination (58); 700-bp regions flanking the gene of interest were amplified for the Δ*zur* (SM89/90, SM91/92), Δ*znuA* (SM107/108, SM109/110), and Δ*znuABC* (SM93/94, SM95/96) mutants and assembled into XbaI-digested pCVD442. Endopeptidase deletion constructs were built as described previously (12). Chromosomal delivery vectors (pJL-1 and pTD101) were used to insert genes via double crossover into native *lacZ*. To construct the *shyB* transcriptional reporter, 500 bp upstream of *shyB* was amplified (SM1/2) and assembled into NheI-digested pAM325 to yield a *P_shyB_*::*lacZ* fusion. This fusion was amplified (SM3/4) and cloned into StuI-digested pJL-1 (59).

10.1128/mBio.02620-18.10 TABLE S2 Summary of oligonucleotides used in this study. Download Table S2, PDF file, 0.08 MB. Copyright © 2019 Murphy et al. 2019 Murphy et al. This content is distributed under the terms of the Creative Commons Attribution 4.0 International license.

To complement gene deletions, *zur* (SM99/100) and *znuA* (SM113/114) were cloned into SmaI-digested pBAD, a chloramphenicol-resistant, arabinose-inducible plasmid. For chromosomal delivery of an IPTG-inducible system into *lacZ*, pTD101(*shyB*) was constructed with SM181/182, and pTD100(*shyA*) was built as previously described (12). An additional chromosomal delivery vector (pSGM100) was built for crossover into VC1807. *shyB* (SM141/SM55) was placed under arabinose-inducible control by cloning into SmaI-digested pSGM100. All assemblies were initially transformed into E. coli DH5ɑ λ*pir* and then into SM10 λ*pir* for conjugation into V. cholerae.

All strains are derivatives of V. cholerae El Tor N16961 (WT), unless otherwise indicated. To conjugate plasmids into V. cholerae, SM10 λ*pir* donor strains carrying pCVD442, pTD101, PJL-1, or pSGM100 plasmids were grown in LB-ampicillin, and strains carrying pBAD were grown in LB-chloramphenicol. Recipient V. cholerae strains were grown overnight in LB-streptomycin. Stationary-phase cells were pelleted by centrifugation (6,500 rpm for 3 min) and washed with fresh LB to remove antibiotics. Equal ratios of donor and recipient (100 µl/100 µl) were mixed and spotted onto LB agar plates. After a 4-h incubation at 37°C, cells were streaked onto LB containing streptomycin and an antibiotic (ampicillin or chloramphenicol) to select for transconjugants. Colonies carrying integration vectors were cured through two rounds of purification on salt-free sucrose (10%) agar with streptomycin. Insertions into native *lacZ* (via pJL-1, pTD101) were identified by blue-white colony screening on X-Gal plates. Insertions into VC1807 were checked via PCR screening (SM/SM). Gene deletions (via pCVD442) were checked via PCR screening with the following primers: Δ*shyA*, TD503/504; Δ*shyB*, SM30/31; Δ*shyC*, TD701/702; Δ*zur*, SM122/123; Δ*znuA*, SM119/120; and Δ*znuABC*, SM119/121).

### Transposon mutagenesis and arbitrary PCR.

The *shyB* transcriptional reporter was mutagenized with Himar1 mariner transposons, which were delivered via conjugation by an SM10 λ*pir* donor strain carrying pSC189 (60). The recipient and donor were grown overnight in LB-streptomycin and LB-ampicillin, respectively. Stationary-phase cells were pelleted by centrifugation (6,500 rpm for 3 min) and washed with fresh LB to remove antibiotics. For each reaction mixture, equal ratios of donor and recipient (500 µl/500 µl) were mixed and spotted onto 0.45-µm filter disks adhered to prewarmed LB plates. After a 4-h incubation at 37°C, cells were harvested by aseptically transferring the filter disks into conical tubes and vortexing in fresh LB. The cells were spread onto LB agar containing streptomycin to kill the donor strain, kanamycin to select for transposon mutants, and X-Gal to allow for blue-white colony screening. Plates were incubated at 30°C overnight, followed by 2 days at room temperature. To identify the transposon insertion site, purified colonies were lysed via boiling and used directly as the DNA template for arbitrary PCR. As described elsewhere, this technique amplifies the DNA sequence adjacent to the transposon insertion site through successive rounds of PCR (24). Amplicons were Sanger sequenced, and high-quality sequencing regions were aligned to the N16961 genome using BLAST (35).

### β-Galactosidase activity measurements.

Strains containing promoter-*lacZ* fusions were grown from single colony in 5 ml of culture medium (LB, M9, or M9 plus 500 μM ZnSO_4_) at 30°C with shaking. Cells from 0.5 ml of exponential-phase culture were harvested, and β-galactosidase assays were performed as described previously (61, 62).

### 5′-Rapid amplification of cDNA ends.

The *shyB* transcription start site was identified with 5′-rapid amplification of cDNA ends (5′-RACE). To obtain a *shyB* transcript, the Δ*zur* mutant was grown in LB at 37°C until cells reached mid-log phase (optical density at 600 nm [OD_600_], 0.5), and RNA was extracted using TRIzol reagent and acid-phenol-chloroform (Ambion). DNA contamination was removed through two RQ1 DNase (Promega) treatments and additional acid-phenol-chloroform extractions. cDNA synthesis was performed with MultiScribe reverse transcriptase (Thermo Fisher) and a *shyB*-specific primer (SM270). cDNA was column purified and treated with terminal transferase (New England BioLabs) to add a homopolymeric cytosine tail to the 3′ end. The cDNA was amplified through two rounds of touchdown PCR with a second gene-specific primer (SM271) and the anchored abridged primer (Thermo Fisher). The PCR product was Sanger sequenced using primer SM271.

### Electrophoretic mobility shift assay.

The LightShift chemiluminescent EMSA kit (Thermo Fisher) was used to detect Zur promoter binding. Forty-one-base pair complementary oligonucleotides (SM264/265) containing the putative *shyB* Zur box, with and without a 5′ biotin label, were annealed according to commercial instructions (Integrated DNA Technologies). Twenty-microliter binding reaction mixtures contained buffer, poly(dI-dC) (50 ng µl^−1^), ZnCl_2_ (5 µM), labeled probe (1 pmol), and purified Zur (600 nM). Unlabeled specific or nonspecific competitor oligonucleotides were added in 100-fold molar excess. Reaction mixtures were incubated on ice for 1 h, electrophoresed on a 6% DNA retardation gel (100 V, 40 min), and wet transferred to a Biodyne B membrane (100 V, 30 min; Thermo Fisher) in a cold room. The membrane was developed using chemiluminescence according to the manufacturer’s instructions and imaged using a Bio-Rad ChemiDoc MP imaging system.

### Protein expression and purification.

DNA encoding N-terminally truncated LysM/M23 endopeptidases (ShyA_Δ1–35_, ShyB_Δ1–34_, and ShyC_Δ1–33_) and full-length Zur was PCR amplified from genomic DNA, while the template for the ShyB H370A mutation was commercially synthesized (Integrated DNA Technologies). Shy constructs were cloned into pCAV4 and Zur cloned into pCAV6, both modified T7 expression vectors that introduce an N-terminal 6×His-NusA tag (pCAV4) or 6×His-MBP tag (pCAV6), followed by a Hrv3C protease site upstream of the inserted sequence. Constructs were transformed into BL21(DE3) cells, grown at 37°C in Terrific broth supplemented with carbenicillin (100 mg ml^−1^) to an OD_600_ of 0.8 to 1.0, and then induced with IPTG (0.3 mM) overnight at 19°C. ZnCl_2_ (50 µM) was added during Zur induction. Cells were harvested via centrifugation, washed with nickel loading buffer (NLB; 20 mM HEPES [pH 7.5], 500 mM NaCl, 30 mM imidazole, 5% [vol/vol] glycerol, 5 mM β-mercaptoethanol), pelleted in 500-ml aliquots, and stored at −80°C.

Pellets were thawed at 37°C and resuspended in NLB supplemented with phenylmethylsulfonyl fluoride (PMSF; 10 mM), DNase (5 mg), MgCl_2_ (5 mM), lysozyme (10 mg ml^−1^), and 1/10 of a cOmplete protease inhibitor cocktail tablet (Roche). All buffers used in Zur purification were supplemented with ZnCl_2_ (1 µM). Cell suspensions were rotated at 4°C, lysed via sonication, and centrifuged, and the supernatant was syringe filtered using a 0.45-µm filter. Clarified samples were loaded onto a NiSO_4_-charged 5-ml HiTrap chelating column (GE Life Sciences) and eluted using an imidazole gradient from 30 mM to 1 M. Hrv3C protease was added to the pooled fractions and dialyzed overnight into cation exchange loading buffer (20 mM HEPES [pH 7.5], 50 mM NaCl, 1 mM EDTA, 5% [vol/vol] glycerol, 1 mM dithiothreitol [DTT]). Cleaved Shy proteins were loaded onto a 5-ml HiTrap Sepharose (SP) high-performance (HP) column, and cleaved Zur was loaded onto a 5-ml HiTrap heparin HP column (GE Life Sciences). All constructs were eluted along a NaCl gradient from 50 mM to 1 M. Fractions were concentrated and injected onto a Superdex 75 16/600 equilibrated in size-exclusion chromatography buffer (20 mM HEPES [pH 7.5], 150 mM KCl, 1 mM DTT). Zur dimers coeluted with maltose-binding protein (MBP) on the sizing column and were subsequently incubated with amylose resin (New England BioLabs) at 4°C, and Zur was collected from a gravity column. Final purified protein concentrations were determined by SDS-PAGE (Fig. S5) and densitometry compared against bovine serum albumin (BSA) standards, as follows: ShyA, 5.72 mg ml^−1^; ShyB, 5.72 mg ml^−1^; ShyB H320A, 2.35 mg ml^−1^; ShyC, 17.93 mg ml^−1^; and Zur, 0.31 mg ml^−1^.

### Sacculi digestion assay and separation by UPLC.

Peptidoglycan from stationary-phase V. cholerae cells was extracted and purified via SDS boiling and digested with muramidase (63). Ten microliters of sacculi and 10 µg of enzyme were mixed in 50 µl buffered solution (50 mM Tris-HCl [pH 7.5], 100 mM NaCl) in the absence or presence of EDTA (0 to 20 mM). Digestions were incubated for 16 h at 37°C, and enzymes were inactivated by boiling during 10 min and centrifugation for 15 min at 22,000 ×* g*. The soluble fraction and the insoluble pellet were separated, and each sample was further digested with muramidase. All soluble products were reduced with sodium borohydride, their pH was adjusted, and they were injected into a Waters UPLC system (MA, USA) equipped with an Acquity UPLC BEH C_18_, 130-Å, 1.7-μm, 2.1 mm by 150 mm column (Waters) and a dual-wavelength absorbance detector. Eluted fragments were separated at 45°C using a linear gradient from buffer A (0.1% [vol/vol] formic acid) to buffer B (0.1% [vol/vol] formic acid, 40% [vol/vol] acetonitrile) in a 12-min run with a flow rate of 0.175 ml min^−1^ and detected at 204 nm. Muropeptide identity was confirmed by MS/MS analysis, using a Xevo G2-XS quadrupole time of flight (Q-TOF) system (Waters Corporation, USA) and the same separation conditions.

### Growth curve analysis.

Strains were grown overnight in LB-streptomycin with IPTG. Cells were washed in 1× phosphate-buffered saline (PBS) and subcultured 1:10 into M9-glucose plus IPTG. After 2 h shaking at 37°C, cells were washed and subcultured 1:100 into M9-glucose containing combinations of TPEN (250 nM), ZnSO_4_ (1 µM), and IPTG (200 µM). The growth of each 200-µl culture in a 100-well plate was monitored by optical density at 600 nm (OD_600_) on a Bioscreen C plate reader (Growth Curves America).

### Microscopy.

Cells were imaged under phase contrast on an agarose patch (0.8% agarose in M9 minimal medium) using a Leica DMi8 inverted microscope.
