# Supplementary figures and images for: Endopeptidase Regulation as a Novel Function of the Zur-Dependent Zinc Starvation Response
Source: mBio. 2019 Feb 19;10(1):e02620-18. doi: 10.1128/mBio.02620-18 (PMC6381278; doi:10.1128/mBio.02620-18)

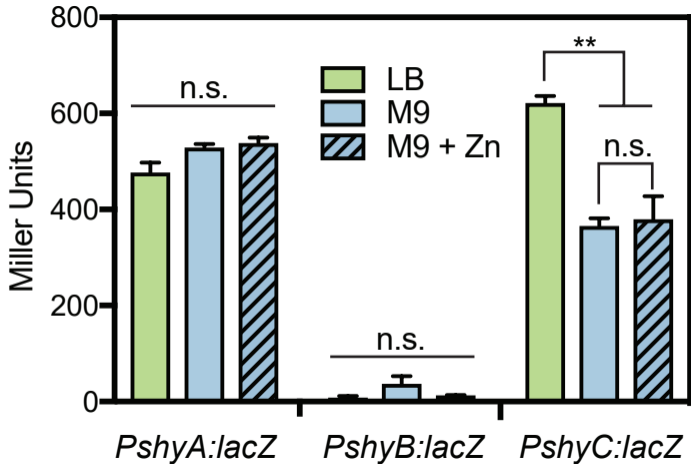

Supplement: FIG S1 [file mBio.02620-18-sf001.pdf]

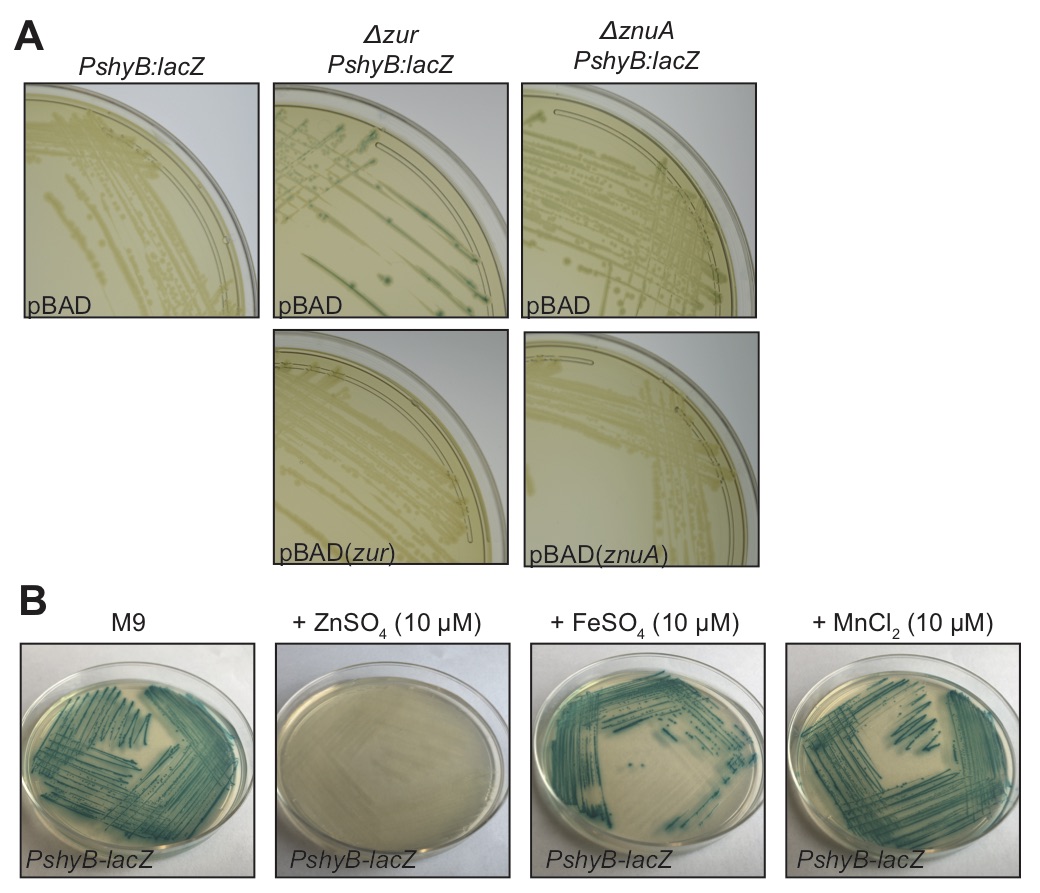

Supplement: FIG S2 [file mBio.02620-18-sf002.jpg]

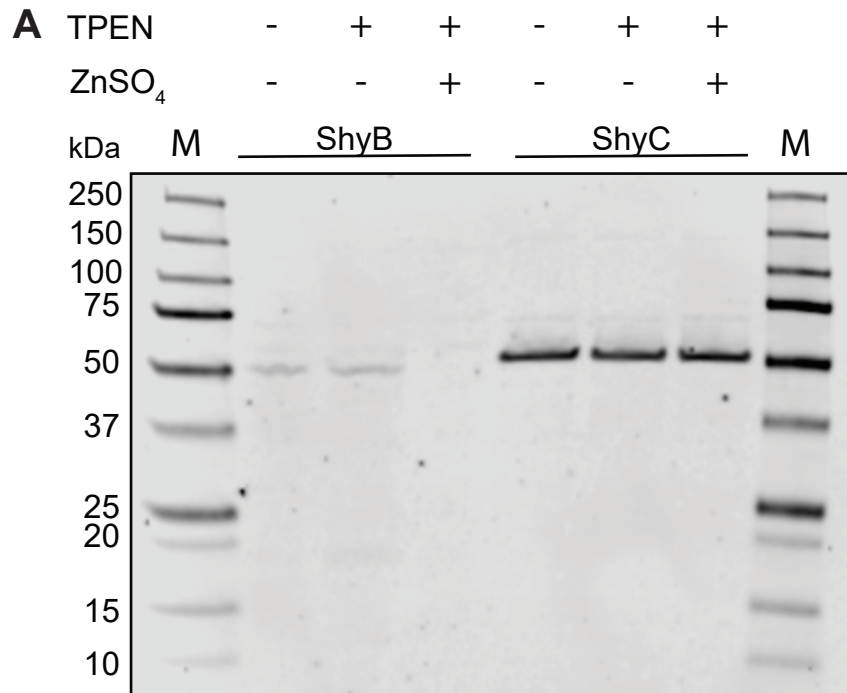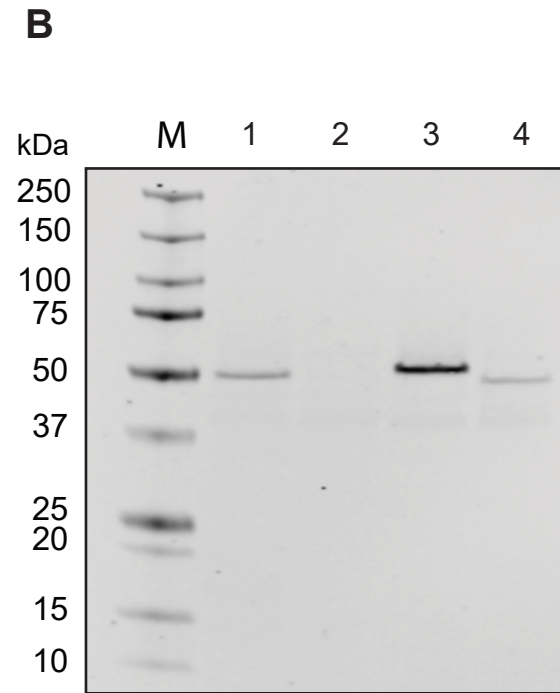

Supplement: FIG S3 [file mBio.02620-18-sf003.pdf]

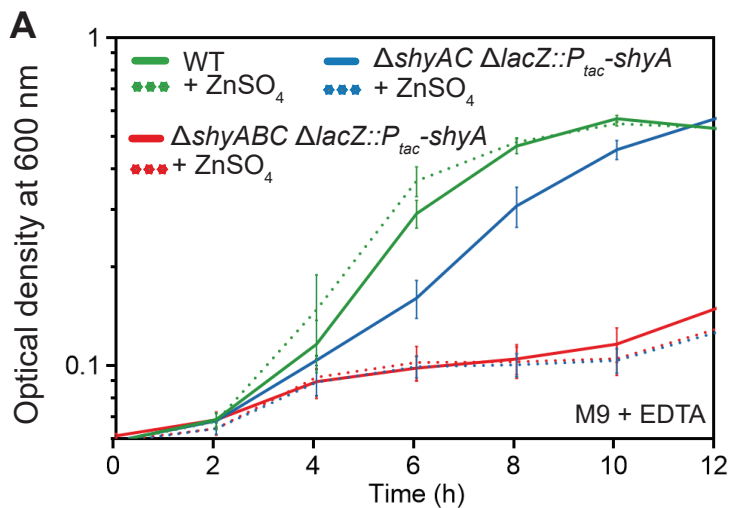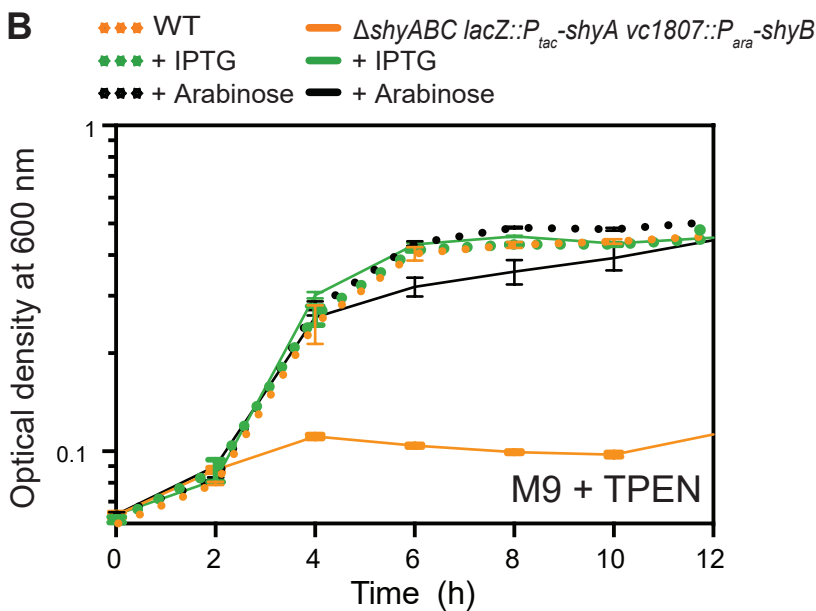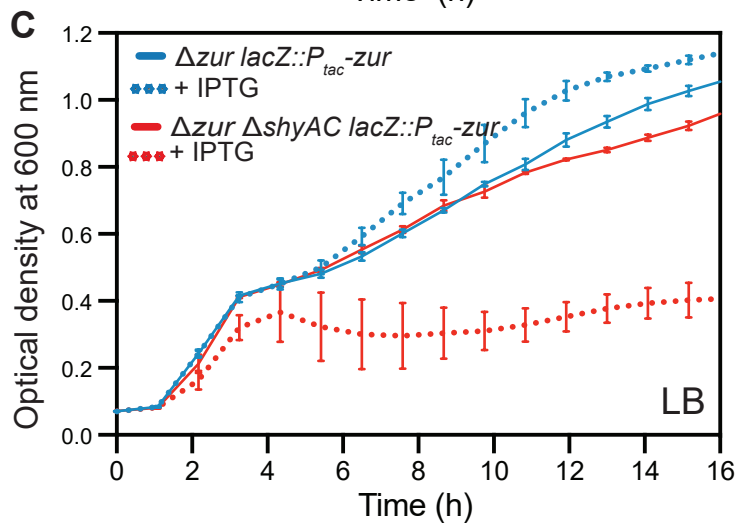

Supplement: FIG S4 [file mBio.02620-18-sf004.pdf]

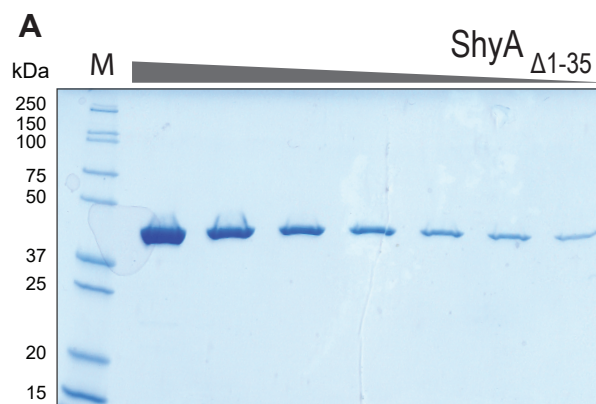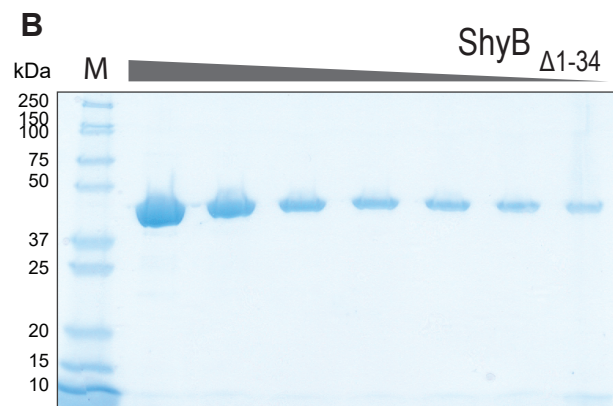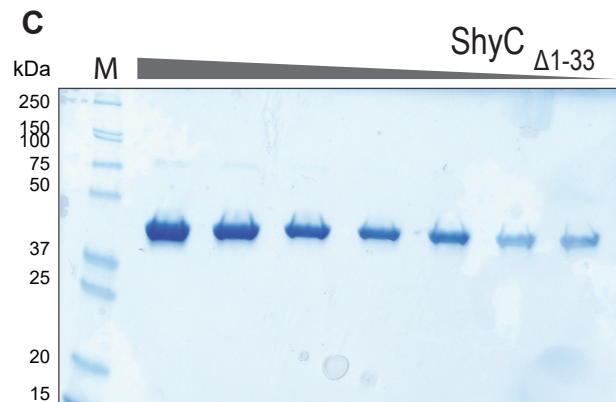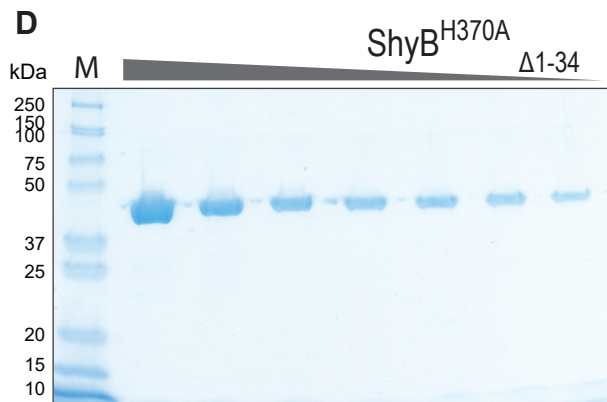

Supplement: FIG S5 [file mBio.02620-18-sf005.pdf]

**A**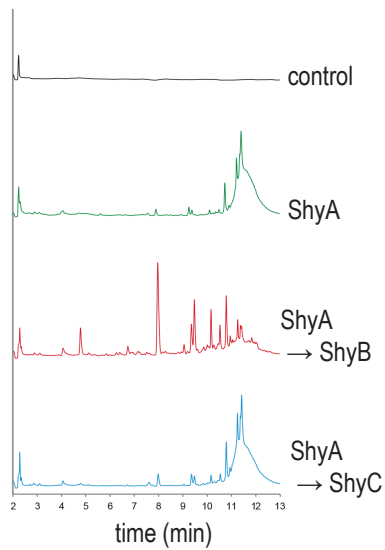**B**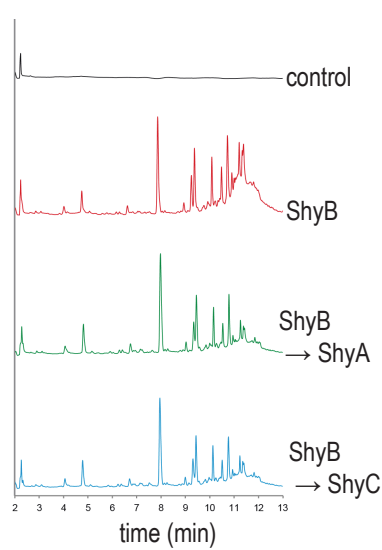**C**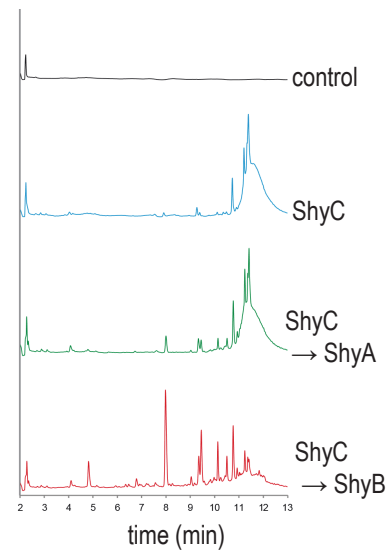**D**

1 hr

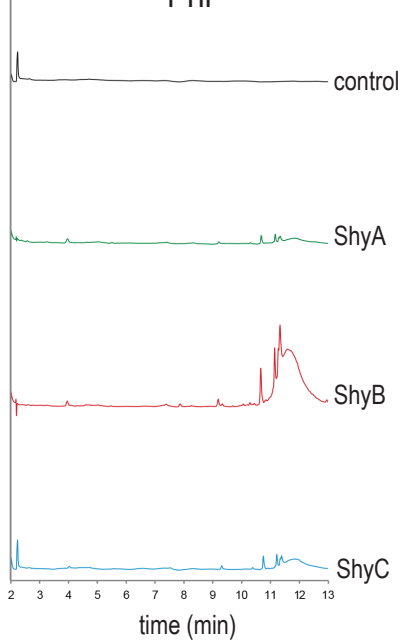**E**

6 hr

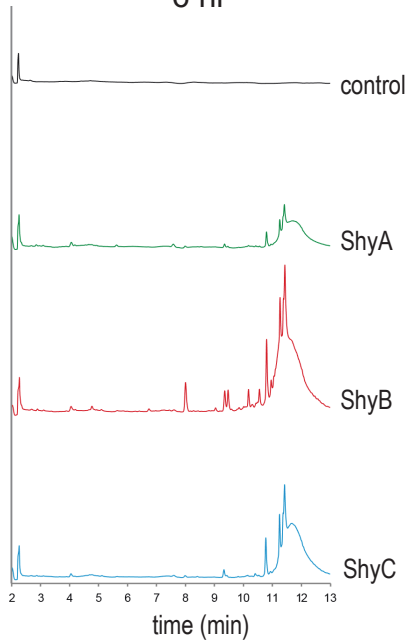**F**

16 hr

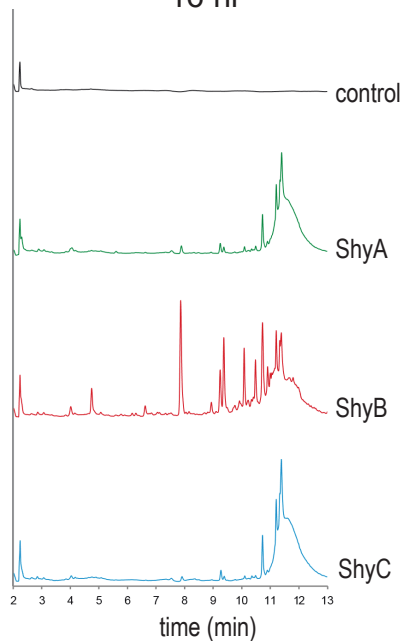

Supplement: FIG S7 [file mBio.02620-18-sf007.pdf]

ShyA

ShyB

ShyC

Soluble fraction

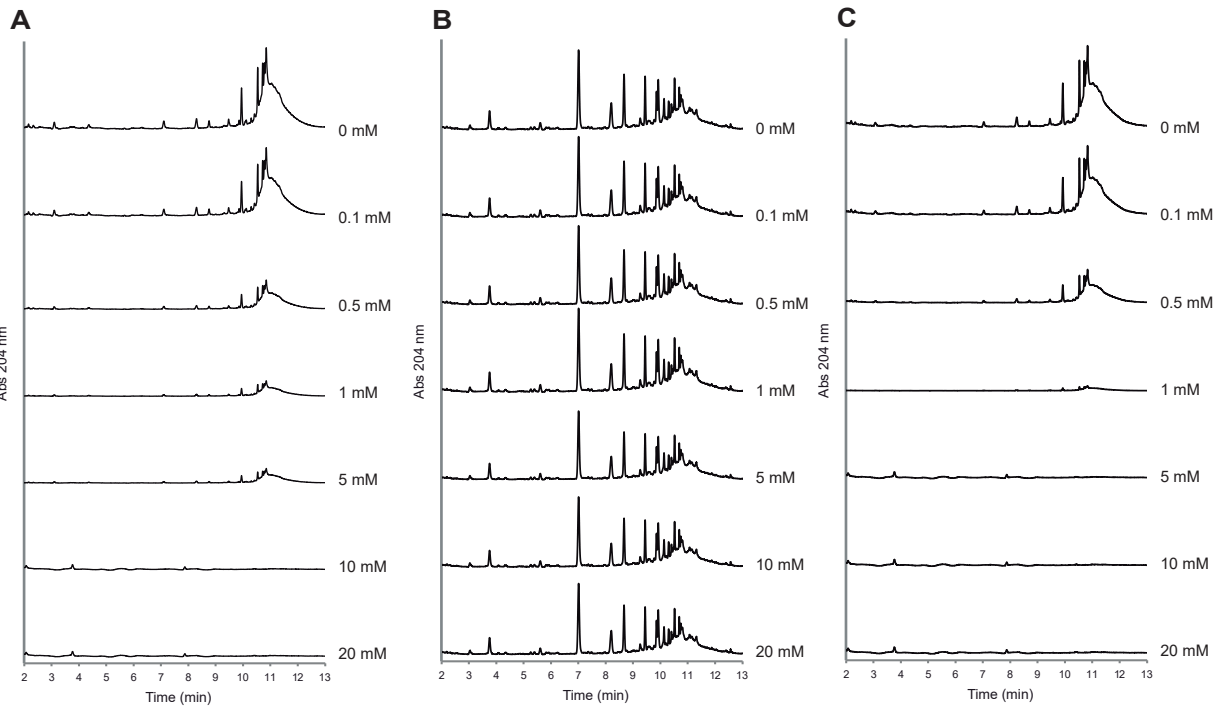

Insoluble fraction + muramidase

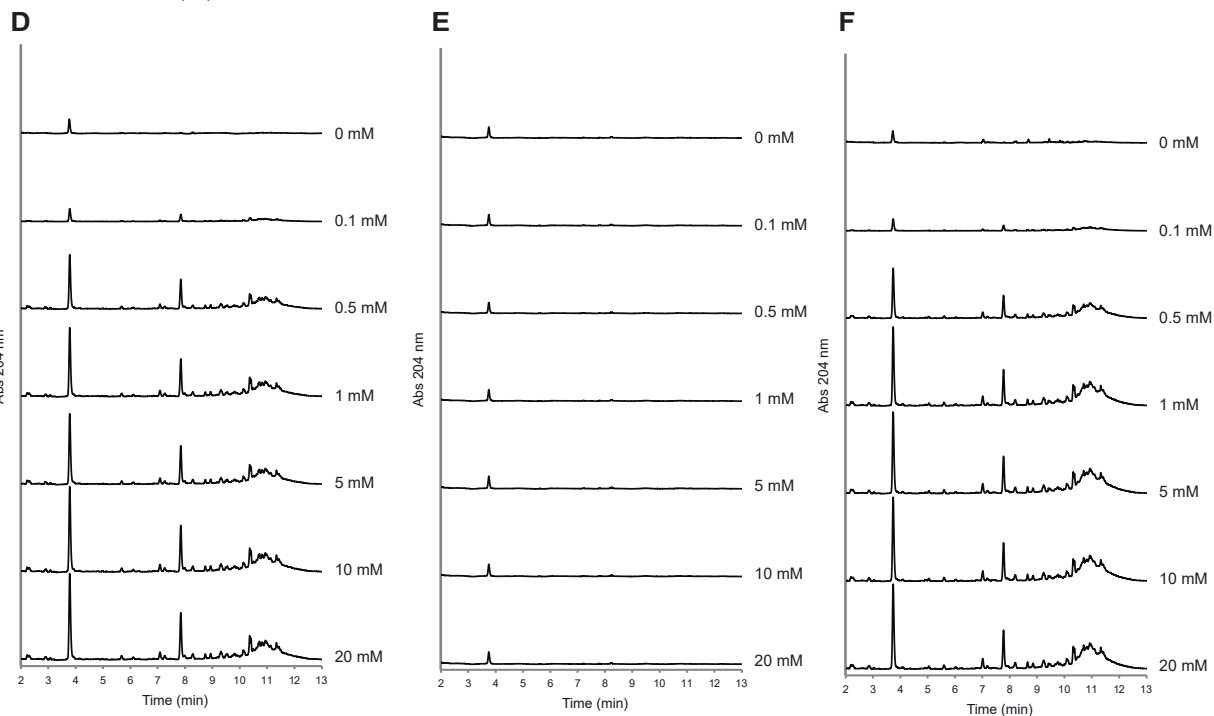

Supplement: FIG S8 [file mBio.02620-18-sf008.pdf]
