# Supplementary material for: Endopeptidase Regulation as a Novel Function of the Zur-Dependent Zinc Starvation Response
Source: mBio. 2019 Feb 19;10(1):e02620-18. doi: 10.1128/mBio.02620-18 (PMC6381278; doi:10.1128/mBio.02620-18)
Supplement: FIG S6 [file mBio.02620-18-sf006.pdf]

A

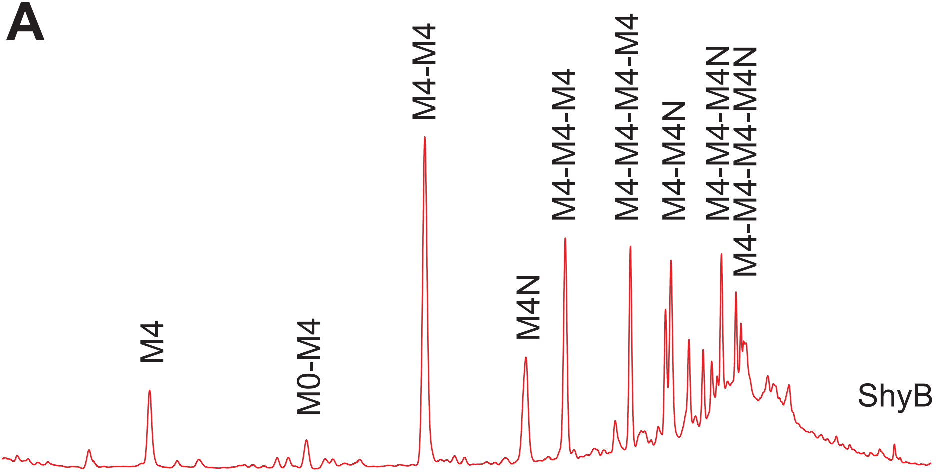

B

Peak	Muropeptide	Monoisotopic mass (g/mol)		Diff.
		Theoretical	Observed	
1	M4	941.408	941.405	0.003
2	M0-M4	1419.588	1419.581	0.006
3	M4-M4	1860.774	1860.753	0.021
4	M4N	921.382	921.377	0.005
5	M4-M4-M4	2782.155	2782.111	0.044
6	M4-M4-M4-M4	3703.537	3703.601	-0.07
7	M4-M4N	1842.763	1842.742	0.021
8	M4-M4-M4N	2764.144	2764.074	0.071
9	M4-M4-M4-M4N	3685.526	3685.478	0.048
