# Supplementary material for: Endopeptidase Regulation as a Novel Function of the Zur-Dependent Zinc Starvation Response
Source: mBio. 2019 Feb 19;10(1):e02620-18. doi: 10.1128/mBio.02620-18 (PMC6381278; doi:10.1128/mBio.02620-18)
Supplement: TABLE S1 [file mBio.02620-18-st001.pdf]

		Homology with ShyB			Putative Zur Box	
**Organism**	**Accession**	Query cover	E value	Identity	Zur box sequence	Distance from ORF (nucleotides)
Vibrio parahaemolyticus	WP_042761124.1	99%	0	91%	tgttataacataaca	29
Vibrio alginolyticus (N646_4535)	KPN02143.1	97%	0	87%	tgttataacataaca	30
Vibrio brasiliensis	WP_040894789	98%	0	68%	tgttatgttataaca	41
Vibrio ponticus	WP_075647776.1	97%	0	64%	tgttatgttataaca	31
Vibrio panuliri	WP_075713859.1	97%	0	62%	tgttatgttataaca	35
Vibrio harveyi	WP_050921596.1	97%	0	60%	tgttataacataaca	31
Vibrio tubiashii	WP_004747324.1	99%	0	61%	tgttataacataaca	26
Vibrio xuii	KOO15045	97%	0	62%	tgttatgttataaca	33
Vibrio breoganii	WP_065209656.1	96%	0	61%	tgttatgttataaca	33
Vibrio rotiferianus	WP_088880208	97%	0	60%	tgttataacataaca	31
Vibrio jasicida	WP_039976478.1	97%	0	59%	tgttataacataaca	31
Vibrio owensii	WP_039989095.1	97%	0	59%	tgttataacataaca	31
Vibrio sinaloensis	WP_008081581.1	96%	0	60%	tgttataacataaca	31
Vibrio campbellii	WP_045454512	99%	1.00E-180	59%	tgttataacataaca	31
Vibrio fortis	WP_032550330.1	98%	1.00E-180	57%	tgttatgttataaca	40
Vibrio maritimus	WP_042471393	99%	5.00E-180	58%	tgttataacataaca	28
Vibrio coralliilyticus	WP_040121393	97%	3.00E-179	59%	tgttatgttataaca	32
Vibrio europaeus	WP_069668665	96%	4.00E-179	60%	tgttataacataaca	26
Vibrio lentus	WP_104211117	96%	9.00E-179	60%	tgttatgttataaca	40
Vibrio hyugaensis	WP_104025248	97%	2.00E-177	58%	tgttataacataaca	31
Vibrio atlanticus	WP_065678199	96%	4.00E-177	59%	tgttatgttataaca	40
Vibrio tasmaniensis	WP_017098377.1	96%	6.00E-177	59%	tgttatgttataaca	40
Vibrio neptunius	WP_045977088	98%	3.00E-176	57%	tgttatgttataaca	33
Vibrio ichthyoenteri	WP_006710590.1	96%	2.00E-176	60%	tgttatgttataacataaca	37
Vibrio splendidus	WP_017075793	98%	2.00E-175	58%	tgttatgttataaca	41
Vibrio cyclitrophicus	WP_010428834	95%	1.00E-173	58%	tgttatgttataaca	41
Vibrio gigantis	WP_086712636	96%	4.00E-173	57%	tgttatgttataaca	39
Vibrio mediterranei	WP_062459131.1	96%	2.00E-169	57%	tgttataacataaca	25
Vibrio crassostrea	WP_017067624.1	97%	4.00E-172	57%	tgttatgttataaca	36
Vibrio chagasii	WP_105023200.1	93%	1.00E-170	58%	tgttatgttataaca	30
