# Supplementary material for: Endopeptidase Regulation as a Novel Function of the Zur-Dependent Zinc Starvation Response
Source: mBio. 2019 Feb 19;10(1):e02620-18. doi: 10.1128/mBio.02620-18 (PMC6381278; doi:10.1128/mBio.02620-18)
Supplement: TABLE S2 [file mBio.02620-18-st002.pdf]

Table S2. Summary of oligonucleotides used in this study.

Primer	Construct	Sequence (5'-3')
SM1	pAM325(*PshyB*)	5'-cagctagcCCAGCTTATTGGCTCAGT-3'
SM2		5'-gtgctagcGAGAACTAAGCATCAATAATTTTGTTATG-3'
SM3	pJL1(*PshyB-lacZ*)	5'-gtgatgattggtacCAGATCTTAATTAAGGCCAGCTTATTGGCTCAGT-3'
SM4		5'-cggggattgGTACCGCGGCCGCTCTAGAGGttattattttgacaccagaccaa-3'
SM89	pCVD442(*Δzur*)	5'-ATATGTGATGGGTTAAAAAGGATCGATCCTGGCTCGCCCATCAGACA-3'
SM90		5'-GCGCATAACCTTCTTTGTTGGACTTTAGTAATAATATACTATGTCGAG-3'
SM91	pCVD442(*Δzur*)	5'-TACTAAAGTCCAACAAAGAAGGTTATGCGCGCTGAAT-3'
SM92		5'-AGAGCTCGATATCGCATGCGGTACCTCTAGCACGCTTGGTGGCAAGGTTTA-3'
SM93	pCVD442(*ΔznuABC*)	5'-ATATGTGATGGGTTAAAAAGGATCGATCCTTGCTGGCACTATTGAGCTGATAAAGT-3'
SM94		5'-GTCGACCAACTTCTCTAACATTGTTCTAAATG-3'
SM95		5'-GAGAAGTTGGTCGACCGCTGTATCAGATTAGCTTTTAGT-3'
SM96		5'-AGAGCTCGATATCGCATGCGGTACCTCTAGGTGACCATCACTGAAGGCATTC-3'
SM107	pCVD442(*ΔznuA*)	5'-ATATGTGATGGGTTAAAAAGGATCGATCCTCTTGCTGGCACTATTGAGCT-3'
SM108		5'-GCCACTAACTGGAGTGTCGACCAACTTCTCTAACATTGT-3'
SM109		5'-GAGAAGTTGGTCGACACTCCAGTTAGTGGCTTTTATTGG-3'
SM110		5'-AGAGCTCGATATCGCATGCGGTACCTCTAGTTGGCGATGCTCTCCGG-3'
SM122	*zur*-colony-Fwd	5'-GATCCTAGTCGGCTTGCCAC-3'
SM123	*zur*-colony-Rev	5'-GAACCGCGCGTCGTTG-3'
SM119	*znu*-colony-Rev	5'-ACTGCGCATCGTTAATCACG-3'
SM120	*zunA*-colony-Fwd	5'-GCGTGTCTCGGCTTCGAT-3'
SM121	*znuABC*-colony-Rev	5'-GCGATCGATGCTGGCATC-3'
SM99	pBAD(*zur*)	5'-tttgggctagcgaattcgagctcggtacccAGGAGGctgactgaGTGATGGTGATGGTTTTGGACAAC-3'
SM100		5'-atgcctgcaggtcgactctagaggatccccTAACCTTCTTCTATTGTTGCTTCTTCTCTTTC-3'
SM113	pBAD(*znuA*)	5'-tttgggctagcgaattcgagctcggtacccAGGAGGctgactgaTTGGCGACCATGTTATCGAGA-3'
SM114		5'-atgcctgcaggtcgactctagaggatccccGTTGGTCGACTTAAGATTGGGAC-3'
SM97	pTD101(*zur*)	5'-aacagaccatggaattcgagctcggtacccAGGAGGctgactgaGTGATGGTGATGGTTTTGGACAAC-3'
SM98		5'-catgcctgcaggtcgactctagaggatccccTAACCTTCTTCTATTGTTGCTTCTTCTCTTTC-3'
SM181	pTD101 (*shyB*)	5'-aacagaccatggaattcgagctcggtacccAGGAGGctgactgaatgGG-3'
SM182		5'-catgcctgcaggtcgactctagaggatccc-3'

SM141	pSGM100(*shyB*)	5'-tttgggctagcgaattcgagctcggtacccAGGAGGTAGTTCTCatgGGTCAATTTAGATTTC-3'
SM55		5'-aacagaccatggaattcgagctcggtacccatgCTTTCTCTTTTCAATCGTCT-3'
SM 270	Arbitrary PCR	5'-AGGTTGAGGTAACGGTGTCA-3'
SM271		5'-GCGACGTGTTAACGCTTTG-3'
SM272	pCAV4 (*shyA*)	5'-AGCGGCCTGGAAGTTCTGTTTCAGGGTCCGCTAAACAGTCCCACGCG-3'
SM273		5'-TTTTTCGAACTGCGGGTGGCTCCAAGCGCTttaTTGCGCTGCTAGCATG-3'
SM172	pCAV4 (*shyB*)	5'-AGCGGCCTGGAAGTTCTGTTTCAGGGTCCGGTTCCACTCAATCAAAGCGTTAACA-3'
SM176		5'-TTTTTCGAACTGCGGGTGGCTCCAAGCGCTttaAGTTTCGTCGAGAGCAACC-3'
SM274	pCAV4 (*shyC*)	5'-AGCGGCCTGGAAGTTCTGTTTCAGGGTCCGGAAGATTTACGTAAACCGGATGC-3'
SM275		5'-TTTTTCGAACTGCGGGTGGCTCCAAGCGCTttaTTGATTGGCATACAGTAACTGG-3'
SM286	pCAV6 (*zur*)	5'-GGCCTGGAAGTTCTGTTTCAGGGTCCGATGGTGATGGTTTTGGACAAC-3'
SM245		5'-TTTTTCGAACTGCGGGTGGCTCCAAGCGCTCTATTGTTGCTTCTTCTCTTTCGA-3'
SM258	vc1807-colony-Fwd	5’- GTCGGTATTCCTCTTGATAAGGATC -3’
SM259	vc1807-colony-Rev	5- GAACATGCTCCATTATGCAAGG -‘3
